# Supplementary material for: Design, Synthesis, and Biological Evaluation of N-Acylhydrazones and Their Activity Against Leishmania amazonensis Promastigotes
Source: Microorganisms. 2025 Jul 2;13(7):1563. doi: 10.3390/microorganisms13071563 (PMC12298860; doi:10.3390/microorganisms13071563)

SERVIÇO DE MÉTODOS ANALÍTICOS

SAMPLE DESCRIPTION: PMM 86 - 17
Registro PDTIS: 121162

INJECTION VOLUME: 10 **SEQUENCE:** C:\CLASS-VP\Sequence\2018\Síntese\João Mafra\CLAE 05 - 20180807.seq **VIAL:** 2
METHOD: C:\CLASS-VP\Methods\2018\Síntese\João Mafra\Cinamicohidrazida - Transposto.met
FILE NAME: C:\CLASS-VP\Data\2018\Sintese\João Mafra\08 - 06\PMM 86 - 17-Rep2
SAMPLE ID: PMM 86 - 17 **ACQUIRED:** 6/8/2018 15:47:49 **PRINTED:** 8/8/2018 09:28:08
ANALISTA: Marcio/Thiago

Sistema de Cromatografia a Líquido: Shimadzu com 2 bombas LC-10AD, degaseificador DGU-12A, injetor automático SIL-10AD, forno de colunas CTO-10A, detector por arranjo de diodos SPD-M10A. Os dados foram adquiridos por interface SCL-10A. Aquisição de dados e controle pelo software Shimadzu CLASS-VP versão 6.13 SP2.

Coluna: Supelcosil C18 150 x 4,6 mm, 3 µm. Código SMA: CL0172
Pré-Coluna: Supelguard C18 - 2cm

Fase Móvel
Eluente (A): Acetato de amônio 0,025 mol/L pH 5,8
Eluente (B): Metanol

Programação:

Time	Module	Command	Value
0,01	Pumps	Pump B	Conc. 5
7,50	Pumps	Pump B	Conc. 5
22,50	Pumps	Pump B	Conc. 95
37,50	Pumps	Pump B	Conc. 95
45,00	Pumps	Pump B	Conc. 5
52,50	Pumps	Pump B	Conc. 5
52,51	Controller	Stop	

Temperatura do forno: 30ºC **Vazão:** 1,0 mL/min

Preparo da amostra: Em uma massa de 0,99 mg, foram adicionados 2,0 mL de metanol agitado com o auxílio de vortex, filtrado em mebrana PVDF de 0,22 µm e injetado.
.

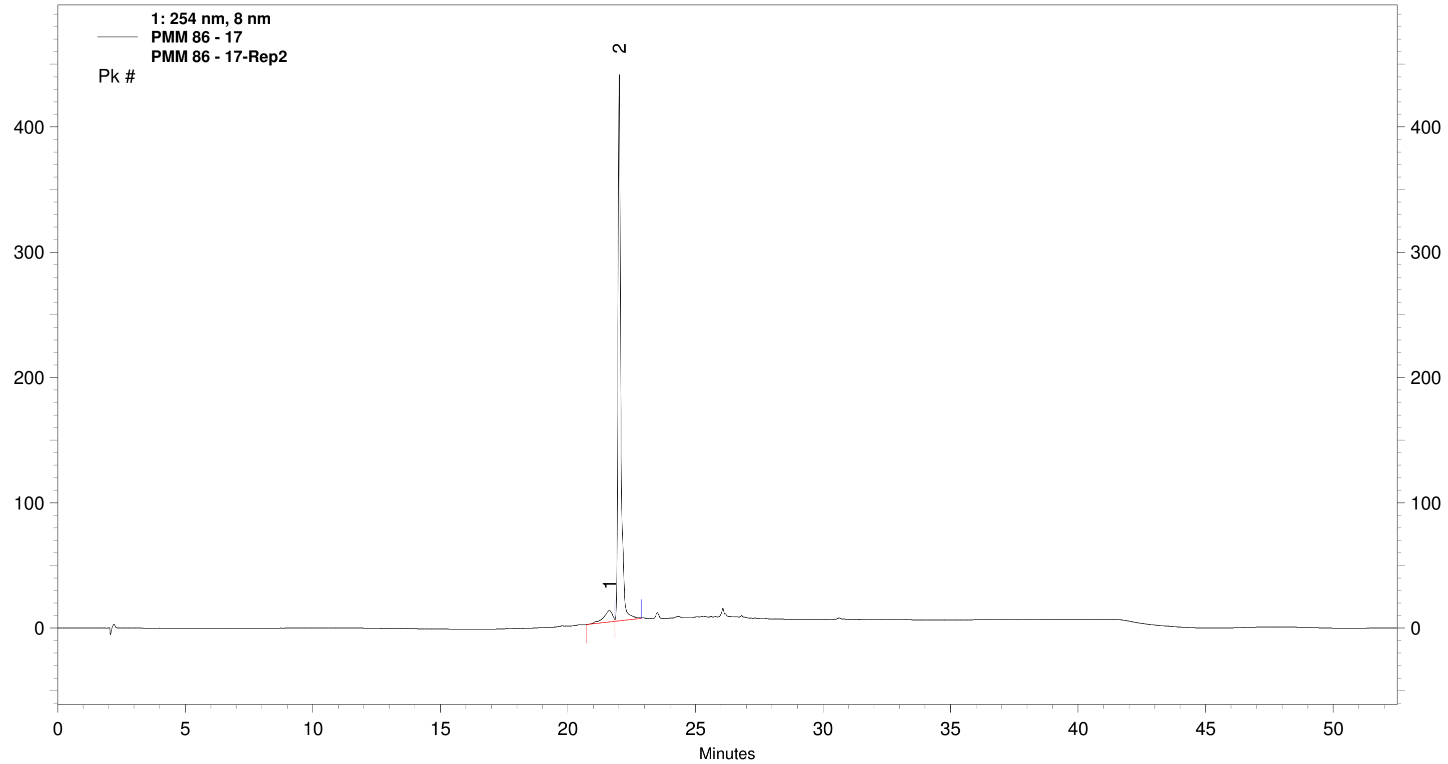

SERVIÇO DE MÉTODOS ANALÍTICOS

SAMPLE DESCRIPTION: PMM 86 - 17
Registro PDTIS: 121162

INJECTION VOLUME: 10 **SEQUENCE:** C:\CLASS-VP\Sequence\2018\Síntese\João Mafra\CLAE 05 - 20180807.seq **VIAL:** 2
METHOD: C:\CLASS-VP\Methods\2018\Síntese\João Mafra\Cinamicohidrazida - Transposto.met
FILE NAME: C:\CLASS-VP\Data\2018\Sintese\João Mafra\08 - 06\PMM 86 - 17-Rep2
SAMPLE ID: PMM 86 - 17 **ACQUIRED:** 6/8/2018 15:47:49 **PRINTED:** 8/8/2018 09:28:08
ANALISTA: Marcio/Thiago

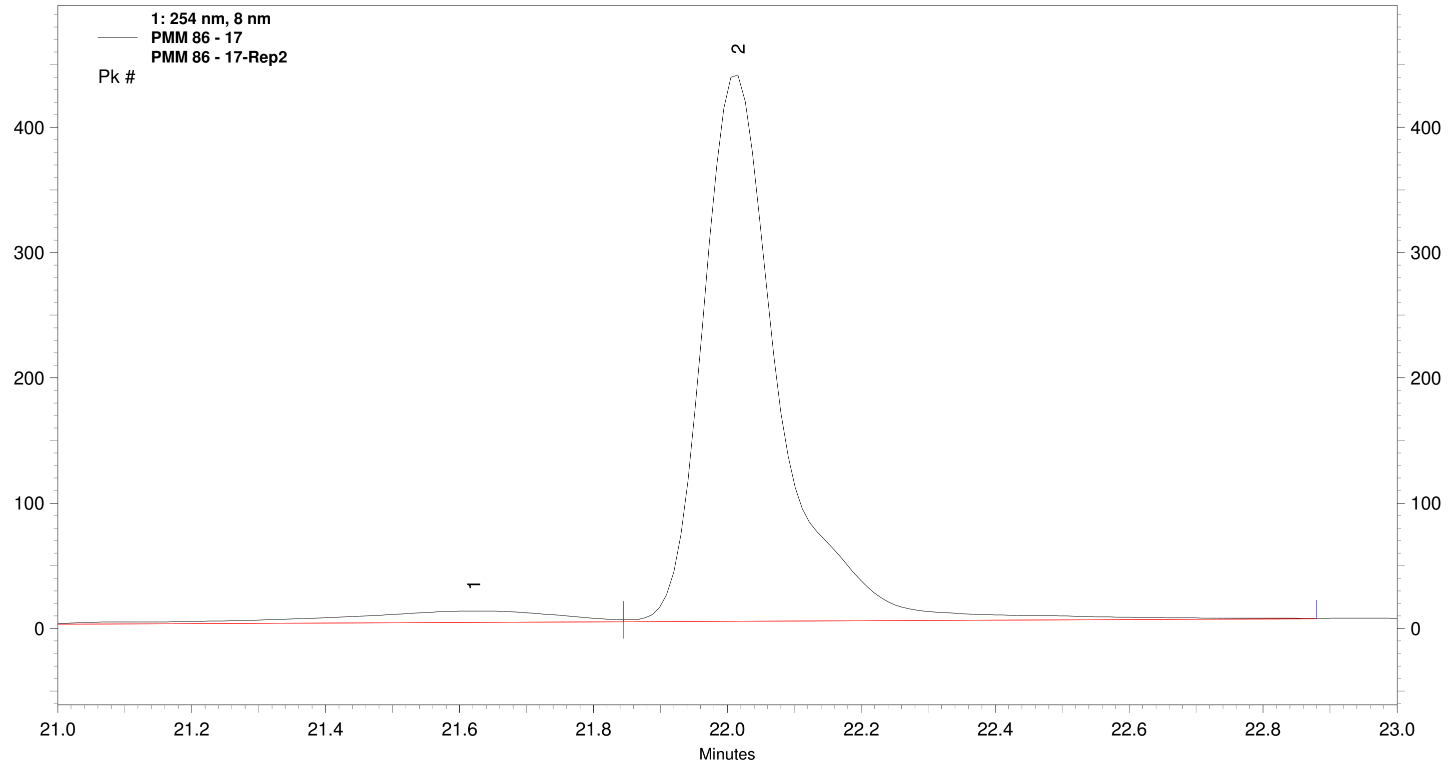

1: 254 nm, 8 nm

Pk #	Retention Time	Area	Area Percent	Asymmetry	Resolution	Peak purity index
1	21.621	220541	5.99	0.62	0.00	0.00000
2	22.016	3463858	94.01	1.42	1.01	1.00000

Totals		3684399	100.00			

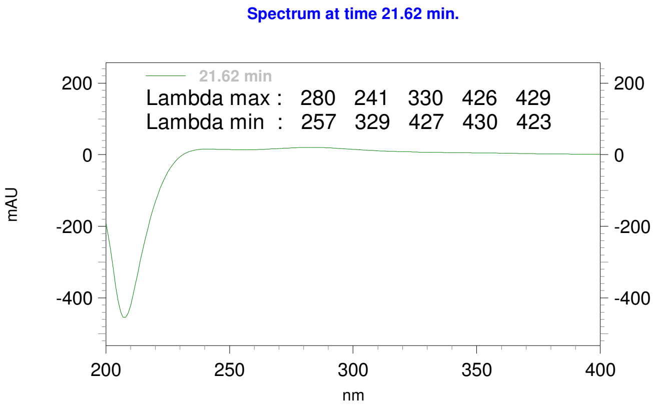

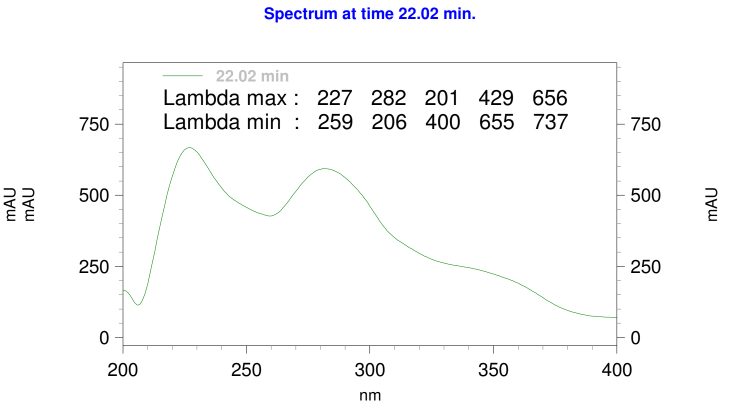

Supplement: Supplementary file 1 [file microorganisms-13-01563-s001.zip › HPLC amostras-20241114T144635Z-001.zip!/HPLC amostras/HPLC composto 18 - PMM 86 - 17.pdf]
